# New Appliances and Things Medical

**Published:** 1903-08-22

**Authors:** 


### NEW APPLIANCES AND THINGS MEDICAL.

[We shall be glad to receive, at our Office, 28 & 29 Southampton Street Strand, London, W.C., from the manufacturers, specimens of all new preparations and appliances which may be brought out from time to time.]

### MALTICO INFANT FOOD.

(TAYLOR AND SONS, KINGSTON CROSS, PORTSMOUTH.)

THIS food is described as suitable for infants who are unable to digest starchy foods with facility. This description is somewhat ambiguous, as every infant up to the age of twelve months is unable to properly cope with starchy food, for the full diastatic action of the pancreatic secretion is not reached till that time. It is easily prepared and palatable, and should be of service as a malted food.

### BOYD'S MALTO-MELLIS.

(MEDICAL AND GENERAL SPECIALITIES COMPANY, 300 CLAPHAM ROAD, LONDON, S.W.)

WE have here a preparation capable of ready absorption and assimilation which should prove of value as an aid to the digestion of farinaceous food, and to delicate children who require a food rich in phosphates. It is highly palatable, and can be readily taken when other foods are rejected.

